# Loss of MHC-I antigen presentation correlated with immune checkpoint blockade tolerance in MAPK inhibitor-resistant melanoma

**DOI:** 10.3389/fphar.2022.928226

**Published:** 2022-08-26

**Authors:** Jing Yu, Xi Wu, Jinen Song, Yujie Zhao, Huifang Li, Min Luo, Xiaowei Liu

**Affiliations:** ^1^ Laboratory of Integrative Medicine, Clinical Research Center for Breast, State Key Laboratory of Biotherapy, West China Hospital, Sichuan University and Collaborative Innovation Center, Chengdu, Sichuan, China; ^2^ Research Core Facility, West China Hospital, Sichuan University, Chengdu, Sichuan, China

**Keywords:** melanoma, MAPK-targeted therapy, immune checkpoint blockade, tumor immune microenvironment, MHC-I antigen presentation, drug resistance (DR)

## Abstract

Immune checkpoint blockade and MAPK-targeted combined therapy is a promising regimen for advanced melanoma patients. However, the clinical benefit from this combo regimen remains limited, especially in patients who acquired resistance to MAPK-targeted therapy. Here, we systematically characterized the immune landscape during MAPK-targeted therapy in patients and mouse melanoma models. We observed that both the abundance of tumor-infiltrated T cells and the expression of immune-related genes were upregulated in the drug-responsive period, but downregulated in the resistance period, implying that acquired drug resistance dampens the antitumor immune response. Further transcriptomic dissection indicated that loss of MHC-I antigen presentation on tumor cells plays a critical role in the reduction of T cell infiltration during drug resistance. Survival analysis demonstrates that loss of antigen presentation and reduction of T-cell infiltration during acquired drug resistance are associated with poorer clinical response and prognosis of anti-PD-1 therapy in melanoma patients. In addition, we identified that alterations in the MAPK inhibitor resistance-related oncogenic signaling pathway closely correlated with deficiency of MHC-I antigen presentation, including activation of the PI3K-mTOR, MAPK, and Wnt pathways. In conclusion, our research illuminates that decreased infiltration of T cells is associated with acquired drug resistance during MAPK-targeted therapy, which may underlie the cross-resistance to immune checkpoint blockade.

## Introduction

The RAS/RAF/MEK/ERK (mitogen-activated protein kinase, MAPK) cascade plays a crucial role in human cancers, with its hyperactivation present in more than 85% of human cancers (Yuan et al., 2020). Many components of the MAPK signaling cascade have been identified as oncogenes, and approximately 8% of cancers harbor BRAF somatic mutations that lead to constitutive activation of downstream signaling of the MAPK cascade (Sanchez-Vega et al., 2018). The frequency of activating BRAF mutations in malignant melanoma cases reaches 50%–60% (Burotto et al., 2014). More than 90% of the BRAF-activating mutations observed in melanoma are single-nucleotide mutations that convert Val 600 to Glu (BRAF^V600E^) (Ascierto et al., 2012). This mutation leads to a conformational change in the BRAF protein, over-activating the downstream kinases MEK1/2-ERK1/2, which in turn activates a series of genes related to cell proliferation and enhanced metabolism, and promotes tumor growth and metastasis (Haling et al., 2014). During the past decade, three specific BRAF^V600E^ kinase inhibitors (BRAFi) and four MEK kinase inhibitors (MEKi) have been approved by the Food and Drug Administration (FDA) for single or dual drug treatment of the BRAF^V600E^ mutated melanomas (Bollag et al., 2010; Bhalla et al., 2011; Flaherty et al., 2012; Roskoski, 2019; Subbiah et al., 2020). Melanoma patients showed an excellent rapid response to MAPK-targeted therapy, with objective response rates (ORRs) ranging from 64% to 87% and median progression-free survival (PFS) ranging from 9.4 to 13.7 months in phase II and III clinical trials (Eroglu and Ribas, 2016). However, approximately 50% of patients experience tumor recurrence 6–8 months after treatment (Chapman et al., 2011; Hauschild et al., 2012). Acquired drug resistance in melanoma severely hinders long-term patient benefit. A long-term anti-tumor response is urgently needed.

Fortunately, immunotherapies, especially the antibody-mediated immune checkpoint blockade (ICB), have shown durable tumor inhibition in the treatment of metastatic melanoma during the past decade year (Hodi et al., 2010; Hamid et al., 2013; Larkin et al., 2015; Robert et al., 2015). Several monoclonal antibodies targeting CTLA-4 and PD-1/PD-L1 have been approved to treat advanced melanoma and various malignant tumors which significantly prolonged patient survival (Hargadon et al., 2018; Bai et al., 2021; Carlino et al., 2021). In a series of clinical trials, nivolumab or pembrolizumab (anti-PD-1 antibody) treatment approached objective overall response rates from 10% to 40% and extended the median overall survival to 10.1 months (Robert et al., 2015; Robert et al., 2021). In a 6-year follow-up study, reliable long-term survival was obtained from anti-PD-1 single-agent treatment in melanoma patients (Hamid et al., 2021; Robert et al., 2021). Nevertheless, only a small subset of patients experiences durable clinical benefits from ICB treatment. Because the therapeutic efficacy of ICB relies on tumor-infiltrating T cells (TILs) to kill tumor cells, patients who lack TILs tend to respond poorly to ICB. Previous studies showed that BRAFi and MEKi treatment could enhance the antigen presentation of melanoma, promote the infiltration of T cells, and induce the expression of PD-L1, thereby modulating the antitumor immune response (Koya et al., 2012; Wilmott et al., 2012; Frederick et al., 2013; Jiang et al., 2013; Knight et al., 2013; Reddy et al., 2016). Meanwhile, data from mouse melanoma models suggest that BRAF and MEK inhibitors can enhance the antitumor activity of immunotherapy (Hu-Lieskovan et al., 2015). A recent study showed that the response to anti-PD-1 immunotherapy inversely correlated with the activation of the MAPK pathway (Fairfax et al., 2020). Based on the complementary advantages and the different action models of the molecular mechanisms of MAPK-targeted therapy and immunotherapy, the combination of MAPK-targeted therapy and ICB is a promising strategy against malignant melanoma.

In the last few years, many triplet therapeutics of BRAF, MEK, and PD-1 or PD-L1 inhibition have been initiated (http://clinicaltrials.gov/). One triple clinical trial, IMspire150 (NCT02908672) (Gutzmer et al., 2020), reported positive data. The use of triple therapy prolonged the median PFS to 15.1 months and the median duration of response to 21.0 months in patients with advanced melanoma. These results suggest that combination with ICB and targeted therapy as first-line therapy for advanced BRAF^V600E^ mutated melanoma can substantially improve progression-free survival, response duration, and overall survival compared with targeted therapy. However, investigator-assessed PFS in patients with BRAF^V600E^ mutated metastatic melanoma in the large phase III clinical trial COMBI-I (NCT02967692) did not show a statistically significant difference between combination regimen and targeted therapy only (Dummer et al., 2022). In addition, in phase II clinical trial KEYNOTE-022 (NCT02130466) (Ferrucci et al., 2020), the median PFS was 16.9 months with triplet and 10.7 months with doublet (HR 0.66, *p* = 0.043). Although the triple arm showed superior benefit, it was negative as it did not meet the study’s prespecified endpoints (HR ≤ 0.62, *p* ≤ 0.025). Conflicting clinical trial results suggest that the combination of targeted therapy and ICB is only effective in some patients. In particular, anti-PD-1/PD-L1 immunotherapy is almost ineffective for BRAFi/MEKi resistant patients (Amini-Adle et al., 2018). Previous research has found that BRAFi/MEKi acquired resistance was associated with the depletion of CD8^+^ T cells in the tumor microenvironment, and BRAFi/MEKi resistance could mediate cross-resistance to anti-PD-1 immunotherapy (Hugo et al., 2015; Hugo et al., 2017). However, the current research on the effect of BRAFi/MEKi on tumor immunity mainly focuses on the treatment response period, ignoring the key changes in the tumor immune microenvironment in the process of drug resistance (Boni et al., 2010; Frederick et al., 2013; Sapkota et al., 2013; Deken et al., 2016). It is difficult to explain the effect of MAPKi on the tumor immune microenvironment throughout the entire treatment period. Therefore, it is very important to comprehensively summarize the dynamic effects of BRAFi and MEKi on the tumor immune microenvironment in the process of targeted therapy, and on this basis, to study the key signals regulating immune changes. This will help explain the specific reasons for the poor response of BRAFi/MEKi and anti-PD-1/PD-L1 combination therapy and provide new ideas for exploring tumor immunity and targeted combination therapy.

In this study, we collected transcriptome data from public databases of baseline, on-treated, and disease-progressed tumor samples from patients with BRAFi or BRAFi + MEKi treatment. Based on the logic that the difference in gene expression before and after treatment in the same patient can genuinely reflect the decisive influence of the drug on it. The immune signatures of the tumor microenvironment at each stage of BRAF/MEK targeted therapy were characterized. The results showed that tumor infiltrated T cells and immune-related genes were upregulated in the response stage and downregulated in the drug-resistant stage. Through bioinformatic analysis, we found that reducing T cell infiltration during the drug resistance process was highly positively correlated with the down-expression of MHC-I antigen presentation on tumor cells. Moreover, we found that the down-expression of MHC-I antigen presentation may be mediated by the re-activating or compensatory activation of oncogenic signaling pathways, such as MAPK, PI3K-mTOR, and Wnt pathway. Our findings lay the groundwork for further elucidation of the mechanisms underlying the cross-resistance of MAPKi-resistant melanomas to immunotherapy and provide new strategies for targeted therapy and immunotherapy in melanoma.

## Materials and methods

### Data collection and preprocessing

Gene expression data for BRAF/MEK-targeted therapy in melanoma patients, including GSE75313, GSE65185, and EGAS00001000992 were obtained from the GEO database and the European Genome-phenome Archive database (Hugo et al., 2015; Kwong et al., 2015; Song et al., 2017), including 39 baselines, 22 on-treated, and 45 disease-progressed tumor samples. A total of 67 paired transcriptome data from pre-treatment, on-treated, and drug-resistant tumor samples in BRAFi (or BRAFi + MEKi) treated melanoma patients were obtained. The on-treated and drug-resistant groups included 22 and 45 pairs of tumor samples, respectively.

Raw data for bulk RNA sequencing of tumor tissue at critical time points of the mouse melanoma cell line SMM102 subcutaneous allograft model in the course of vemurafenib treatment were obtained from the GEO (GSE161430, *n* = 14) database (Xu et al., 2022). Gene expression profiling data were obtained by aligning sequencing data to mouse reference genome GENCODE GRCm38 using the Kallisto (v0.42.6) (Bray et al., 2016).

Mouse melanoma single-cell transcriptomic gene expression data were obtained through the GEO database (GSE126714), which contains single-cell transcriptomic data from 618 tumor cells, 222 T/NK cells, 794 macrophages, and 762 monocytes in the BRAF-targeted therapy before treatment, during the response, and the drug resistance (Long et al., 2019). Single-cell data were analyzed and visualized using Seurat (v3.1.2) as described in our previously published article (Stuart et al., 2019; Zheng et al., 2022).

Sequencing data from pre-treatment tumor biopsies of melanoma patients receiving anti-PD-1 monotherapy was downloaded from the European Nucleotide Archive (PRJEB23709, *n* = 41) database (Gide et al., 2019). To obtain the gene expression data, the downloaded sequencing data were aligned to the human reference genome GENCODE GRCh38 by Kallisto. The clinical information corresponding to the data is available in a recent publication by Gide et al. (2019).

### Evaluation of tumor immune infiltration

For expression data from biopsy tissues of MAPK-targeted therapy and PD-1 immunotherapy with melanoma patients (GSE75313, GSE65185, EGAS00001000992, and PRJEB23709), we analyzed their tumor infiltration using microenvironment cell population (MCP) -counter algorithm, which allows quantification of the absolute abundance of immune and stromal cell populations in heterogeneous tissues (Becht et al., 2016). Cytotoxicity scores are also calculated by MCP-counter software according to the gene expression profile of the samples. For the bulk transcriptomic data from the mouse melanoma model (GSE161430), the tissue-infiltrating immune and stromal cells were quantified using murine Microenvironment Cell Population counter (mMCP-counter) (Petitprez et al., 2020).

### Differential expression analysis and functional annotation of differentially expressed genes

To find the kinetics of dynamic changes in the tumor microenvironment during melanoma MAPK-targeted therapy, we performed differential expression analysis of biopsy samples during the response and resistant phases. Since these samples were derived from three datasets, we first used additive model formulas of limma (v3.44.3) to remove batch effects existing between different datasets and then performed differential expression analysis using limma (v3.44.3) (Ritchie et al., 2015). Our criteria for screening differentially expressed genes (DEGs) were as follows: the absolute value of log2FC >1, and adjusted *p*-value < 0.05. We then annotated the functions of these DEGs using clusterProfiler (v3.16.1) (Wu et al., 2021), and the pathway for enrichment analysis was obtained from the Kyoto Encyclopedia of Genes and Genomics (KEGG) database by KEGGREST (v1.28.0). When the adjusted *p*-value < 0.05, we considered this pathway to be significantly enriched. The KEGG BRITE functional-hierarchies information was downloaded from the KEGG database (https://www.kegg.jp).

### Gene set variation analysis

GSVA is a non-parametric and unsupervised method, which can calculate sample-wise gene set enrichment scores as a function of genes inside and outside the gene set (Hänzelmann et al., 2013). Therefore, we scored gene set activity in different samples using GSVA (v1.36.3). The predefined Gene Ontology (GO)-Biological Processes (BP) reference gene sets were derived from the Molecular Signatures Database (MsigDB) (Liberzon et al., 2011). We defined the “GOBP_ANTIGEN_PROCESSING_AND_PRESENTATION_OF_ENDOGENOUS_PEPTIDE_ANTIGEN” and “GOBP_ANTIGEN_PROCESSING_AND_PRESENTATION_OF_PEPTIDE_OR_POLYSACCHARIDE_ANTIGEN_VIA_MHC_CLASS_II” gene sets as the MHC-I and MHC-II antigen presentation pathways, respectively, according to the standard name and included genes.

### Gene set enrichment analysis

To assess the relationship between pathway and phenotype, we performed GSEA analysis using clusterProfiler (v3.16.1) on biopsy samples with different phenotypes of MAPK-targeted therapy for melanoma. Genes were ordered according to fold change. At adjusted *p*-value < 0.05, we identified this pathway with differential activity across phenotypes. In this functional analysis, the required gmt file was generated by the KEGGREST software, which contains all human KEGG pathways in the Kyoto Encyclopedia of Genes and Genomes database.

### Immunohistochemistry and immunofluorescence

To evaluate the changes in the abundance and function of tumor-specific T cells in tumor tissues during MAPK-targeted therapy. We detected the number of CD8^+^ T cells and GZMB^+^ T cells in tumor tissue by immunofluorescence and immunohistochemistry, respectively. The slides were derived from our previously collected BRAFi-treated mouse melanoma tumors (Xu et al., 2022). The immunofluorescence and immunohistochemistry were conducted according to our previous study (Liu et al., 2019; Liu et al., 2021). Briefly, tumor tissues were first fixed in 10% formalin, and then paraffin-embedded, and 4 μm thick tumor sections were mounted on slides for staining. Slides were deparaffinized and incubated in 3% hydrogen peroxide, followed by antigen retrieval in EDTA (pH = 9.0) for 3 min in a pressure cooker. After rinsing with PBS at room temperature, slides were incubated with CD8 (CST, 1:500, #98941) and GZMB (CST, 1:100, #44153) primary antibody overnight. For GZMB immunohistochemistry, slides were washed with PBS and then conjugated to the appropriate horseradish peroxidase-conjugated secondary antibody for 30 min at room temperature. Finally, the slides were incubated with 3,3′-diaminobenzidine (DAB) for visualization. For CD8 immunofluorescence, slides were washed with PBS and then conjugated to the fluorescence-conjugated secondary antibodies at room temperature for 1 h (protected from light). Finally, slides were stained with DAPI nuclear dye for visualization.

To calculate the cell densities of CD8^+^ T cells and GZMB^+^ T cells, we stained five tumor tissues from each group with anti-CD8 and anti-GZMB antibody and captured five representative non-overlapping fields at ×200 magnification by Olympus cellSens. Then, the images were analyzed by ImageJ software to calculate the cell density (number of positive cells/mm^2^).

### Survival analysis

To explore the effect of pathway activity and gene expression level on the prognosis of melanoma anti-PD-1, we analyzed patient survival and prognosis using survival (v3.2-7) and survminer (v0.4.8). A total of 41 patients were included in the analysis, and the data were grouped using the optimal cutoff point. The optimal cutoff point was defined as the most significant segmentation point (with minimal *p*-value). The Kaplan-Meier method was used to plot the survival curves, and the statistical significance was assessed by the log-rank test. The criterion for a statistically significant difference was *p*-value < 0.05.

### Statistical analysis

If not specifically stated, the relevant data analysis in this study was performed in the R program (v4.0.3). The Wilcoxon test was used to compare continuous data. Pearson’s correlation was used to assess the correlation. For survival analysis, log-rank test was used for statistical analysis between Kaplan-Meler curves. *p*-value was usually corrected with a multiple hypothesis test using Benjamini-Hochberg (BH). The results were presented as mean ± SD. We use the following convention for symbols indicating statistical significance: ^ns^ *p* > 0.05; *p < 0.05; **p < 0.01; ***p < 0.001; ****p < 0.0001.

## Results

### Acquired MAPKi resistance reduces tumor-infiltrating T cells

**FIGURE 1:**
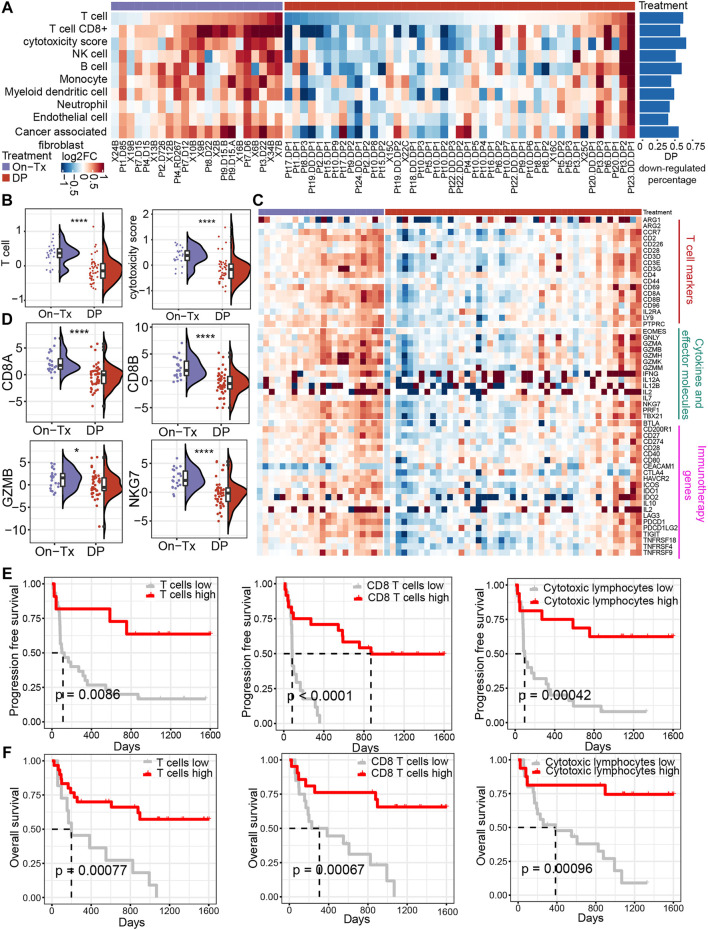
Dynamic changes in the immune microenvironment during MAPK-targeted therapy in melanoma. **(A)** Heatmap of log2FC expression in tumor-infiltrating immune cells and stromal cells between MAPKi-treated and patient-matched baseline biopsies (GSE75313, GSE65185, and EGAS00001000992). The rows have been sorted according to the log2FC of the T cell. The right histogram shows the proportion of samples with downregulated infiltrating cell abundance in the resistant biopsies compared to the baseline biopsies **(B)** Boxplot showing differences in log2FC expression of T cell and cytotoxicity scores between on-treatment and resistance biopsies. The log2FC was obtained by comparison with patient-matched baseline biopsy samples. Statistical analysis was performed using the Wilcoxon test. **(C)** The heatmap shows the changes in the expression of T cell marker genes, cytokines and effector molecule genes, and immunotherapy-related genes in the treated biopsies relative to baseline biopsies during MAPKi treatment. The order of the rows is consistent with that shown in **(A)**. **(D)** Boxplot showing differences in log2FC expression of CD8A, CD8B, GZMB, and NKG7 between on-treatment and resistance biopsies. The log2FC was obtained by comparison with patient-matched baseline biopsy samples. Statistical analysis was performed using the Wilcoxon test. **(E)** Kaplan-Meier plots showing progression-free survival (PFS) of anti-PD1 treated melanoma patients, stratified by using the optimal cutoffs for T cell, CD8^+^ T cell, and cytotoxic lymphocyte infiltrating abundance (PRJEB23709). The optimal cutoff is defined as the point with the smallest *p*-value (log-rank test) split. **(F)** Kaplan-Meier plots showing overall survival (OS) of anti-PD1 treated melanoma patients, stratified by using the optimal cutoffs for T cell, CD8^+^ T cell, and cytotoxic lymphocyte infiltrating abundance (PRJEB23709). The optimal cutoff is defined as the point with the smallest *p*-value (log-rank test) split. ∗*p* < 0.05; ∗∗*p* < 0.01; ∗∗*p* < 0.001; *∗∗∗*p* < 0.0001. On-Tx: biopsies on-treatment of MAPKi; DP: biopsies resistant with MAPKi.

To evaluate the influence of MAPK-targeted therapy on the tumor immune environment of patients, we re-analyzed the transcriptome data from baseline (before treatment), on-treated (On-Tx, drug-responsive period), and the disease progressed (DP, drug resistance period) melanoma biopsies, which were treated with BRAFi or BRAFi plus MEKi (Hugo et al., 2015; Kwong et al., 2015; Song et al., 2017). We presume that the influence of the targeted drug on tumor immune response could be assessed by comparing the treated versus matched baseline tumors in the light of immune response-related terms. We identified tumor tissue-infiltrating cell types from transcriptomic data using MCP-counter (Becht et al., 2016). The results showed that the tumor immune microenvironment changes dynamically during the MAPK-targeted therapy. In the response period, MAPK-targeted therapy can increase the infiltration abundance of T cells and enhance their function compared to the patient-matched baseline biopsies. In the drug resistance period, the infiltration and function of T cells were significantly inhibited (Figures 1A,B). We observed that 60% of MAPKi-resistant melanomas displayed a relative loss of T cells and their function. (Figure 1A). The changing trend of T cell marker genes, cytokines and effector molecule genes during MAPK-targeted therapy was consistent with T cell infiltration abundance changes. Indeed, the abundance of infiltrating T cells and the expression of immune-related genes were upregulated in on-treatment tumors and downregulated in disease-progression tumors (Figures 1C,D). In addition, by analyzing the expression of genes currently targeted for cancer immunotherapy in clinic, we found that the expression of those genes was downregulated in the MAPKi-resistant group (Figure 1C).

To illustrate the relationship between the changes in the abundance of T cell infiltration observed above and the clinical response to ICB therapy. By performing a survival analysis in melanoma patients receiving anti-PD-1 treatment (*n* = 41) (Gide et al., 2019), we found that patients with higher T cell infiltrating abundance and more active T cell functional status could achieve longer PFS and overall survival (OS) in anti-PD-1 therapy (Figures 1E,F). The observations suggest that the abundance of tumor-infiltrating T cells changes markedly with the response stage during MPAK-targeted therapy. The main manifestations are high levels of T-cell infiltration during the drug response period and a marked downregulation of infiltrating abundance during disease progression. Tumor-infiltrating T cells play a critical role in the clinical response to ICB therapy, and their increased abundance may improve the prognosis of PD-1 blockade therapy.

### Dynamic changes of immune signature in MAPKi-resistant melanoma were reproduced and confirmed in mouse model

**FIGURE 2:**
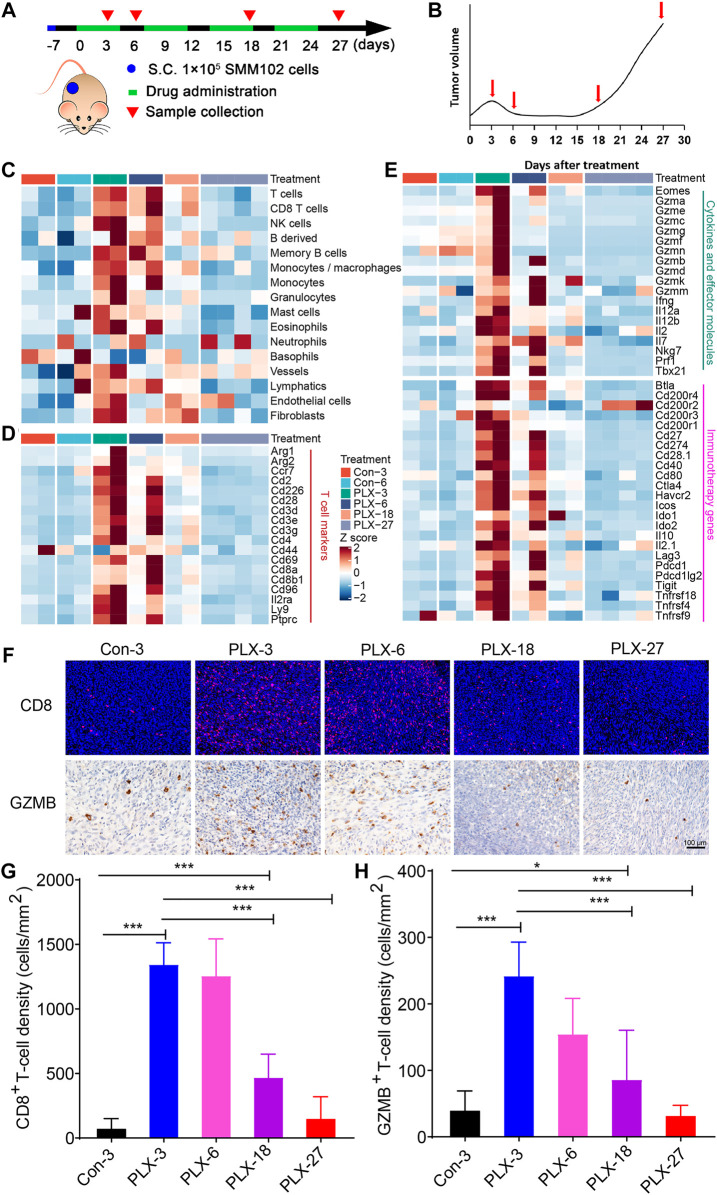
Dynamic changes in immune signatures during BRAFi treatment in the mouse melanoma models. **(A)** Schematic representation of the experimental design used for the mouse melanoma model with or without BRAFi treatment. On day -7, 1 × 10^5 SMM102 tumor cells were inoculated on the backs of C57BL/6 mice, and the tumor-bearing mice were treated with saline (control), or vemurafenib (PLX4032) for the corresponding times and analyzed at the indicated time points (day 3, 6, 18, and 27). **(B)** The resistance curve model of SMM102 tumor to vemurafenib. **(C)** Dynamic changes in tumor-infiltrating lymphocyte and stromal cell abundance at different time points of BRAFi treatment in the mouse melanoma models (GSE161430). **(D,E)** Heatmap showing changes in the expression levels of T cell marker genes, cytokines and effector molecule genes, and immunotherapy target genes at different times of BRAFi treatment in the mouse melanoma model. **(F)** Anti-CD8 immunofluorescence and anti-GZMB immunohistochemistry staining of BRAFi-treated mouse melanoma tissues. **(G,H)** Quantification of CD8^+^ cells and GZMB^+^ cells. The results are presented as the mean ± SD (*n* = 5), and statistical tests were performed using one-way ANOVA. ∗*p* < 0.05; ∗∗*p* < 0.01; ∗∗∗*p* < 0.001; ∗∗∗∗*p* < 0.0001.

Due to the ethical limitations of clinical samples (the inflexible sample collection), we cannot accurately characterize the changes in the tumor immune microenvironment at indicated time points during MAPK-targeted therapy. To further validate the above observations in melanoma patients, we replayed the loss of immune signaling during BRAFi treatment in a mouse model. In our previous study, a xenograft mouse melanoma model was established by SMM102 mouse melanoma cells, and the mice were treated with a BRAFi (vemurafenib, PLX4032) (Xu et al., 2022) (Figure 2A). Based on the tumor growth curves (Figure 2B), the evolution of drug resistance was defined as four stages: 1) early responsive period (day 3, PLX-3); 2) persistent period (day 6, PLX-6); 3) early drug resistance period (day 18, PLX-18); and 4) stable drug resistance period (day 27, PLX-27). We used mMCP-counter to assess the tumor microenvironment based on the RNA sequence data from either untreated (Con-3/6) or BRAFi-treated melanoma (PLX-3/6/18/27) (Petitprez et al., 2020). The results showed that BRAFi treatment promoted the intra-tumoral T cells infiltration and enhanced their function in both the responsive period and early drug resistance period (PLX-3/6/18). However, in the stable drug resistance period (PLX-27), the number of tumor-infiltrating T cells was reduced (Figure 2C). Other immune-related genes also showed the same trends, including T cell marker genes, cytokines, and effector molecule genes, and cancer immunotherapy target genes (Figures 2D,E). This is consistent with what we observed in clinical biopsy samples.

We then detected the number and function of tumor-infiltrating CD8^+^ T cells by immunohistochemistry and immunofluorescence (Figure 2F). The results showed that from the early responsive period (PLX-3) to the early drug resistance period (PLX-18), CD8^+^ T cell infiltration was significantly increased compared to the untreated group (Figures 2F,G). In the stable drug resistance period (PLX-27), the abundance of tumor-infiltrating CD8^+^ T cells was significantly decreased (Figure 2F). Simultaneously, the protein level of GZMB, a functional marker of T cells, during BRAF-targeted therapy also showed the same trend as CD8^+^ T cells (Figures 2F,H). These results indicates that during MAPKi-targeted therapy, the abundance of tumor-infiltrating T cells undergoes corresponding dynamic changes at different stages of treatment (response stage, drug resistance stage), and the regulatory effect of BRAFi/MEKi on the immune microenvironment presents staged changes.

### Identifying the potential regulatory pathway of T cell infiltration during targeted therapy

**FIGURE 3:**
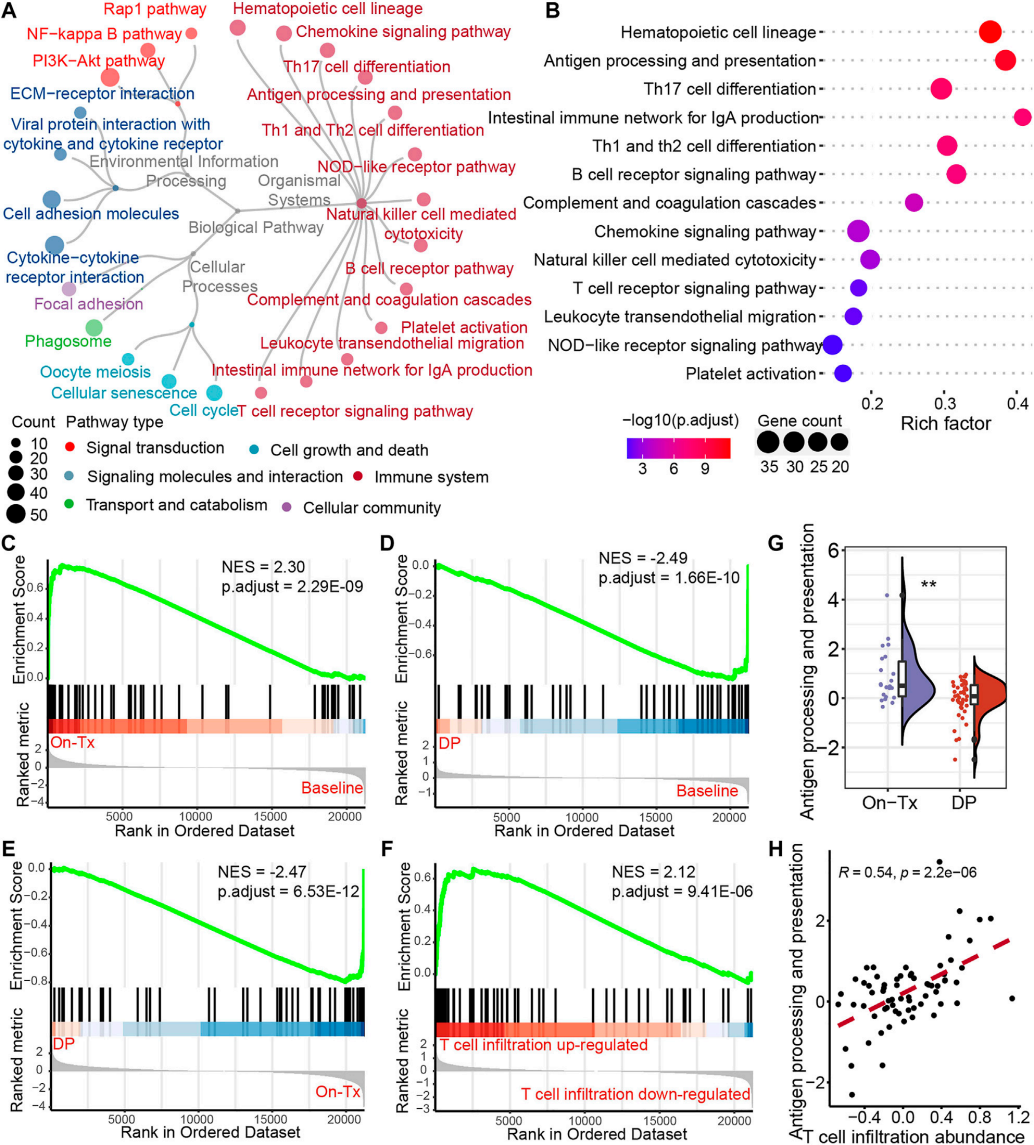
Potential regulatory pathways of T-cell infiltration during MAPK-targeted therapy. **(A)** The circular dendrogram shows the KEGG pathways that were significantly enriched (adjusted *p*-value < 0.05) for functional annotation using differentially expressed genes between On-Tx and DP biopsies (GSE75313, GSE65185, and EGAS00001000992). **(B)** The figure shows all the pathways belonging to the immune system in **(A)**. **(C–E)** Gene Set Enrichment Analysis (GSEA) showing antigen processing and presentation pathway activity at different stages of MAPK-targeted therapy, **(C)** between On-Tx and Baseline biopsies **(D)** between DP and Baseline biopsies, **(E)** between DP and On-Tx biopsies. **(F)** GSEA showing antigen processing and presentation pathway activity between resistance biopsies with up- and downregulated T cell infiltration abundance relative to baseline biopsies. **(G)** Boxplot showing differences in log2FC expression of antigen processing and presentation pathway between on-treatment and drug-resistance biopsies. The log2FC was obtained by comparison with patient-matched baseline biopsy samples. Statistical analysis was performed using the Wilcoxon test. **(H)** Scatter plot showing Pearson’s correlation between antigen processing and presentation pathway and abundance of T cell infiltration. The value used to calculate the correlation was log2FC expression between MAPKi-treated and patient-matched baseline biopsies. ∗∗*p* < 0.01. NES normalized enrichment score.

The efficacy of anti-PD-1/PD-L1 immunotherapy is closely related to the immune status in the tumor microenvironment and depends on tumor antigen-specific cytotoxic CD8^+^ T lymphocytes. To investigate the mechanism of dynamic T cell infiltration during MAPK-targeted therapy, we analyzed differentially expressed genes at different stages of MAPKi-resistant using transcriptomic data from melanoma patients. The analysis results showed that 526 significantly upregulated and 1,037 significantly downregulated genes were identified in the drug resistance biopsies compared to the on-treatment biopsies (absolute value of log2FC > 1 and adjusted *p*-value < 0.05). To further investigate the potential biological functions of these DEGs, we performed the enrichment analysis based on the Kyoto Encyclopedia of Genes and Genomes (KEGG) database. A total of 25 pathways were significantly enriched (adjusted *p*-value < 0.05). According to the KEGG BRITE functional-hierarchies tree, the enriched pathways can be categorized into six classes, and more than half of these pathways belong to the immune-related pathway (Figure 3A). Furthermore, we also found the enrichment of drug resistance-related signal transduction pathways, such as Rap1, NF-kappaB, and PI3K-AKT signaling pathways. Importantly, we found the antigen processing and presentation pathway was ranked highly by ranking these immune system-related pathways according to the adjusted *p*-value (Figure 3B). Furthermore, gene set enrichment analysis (GSEA) found that the activity of the antigen presentation pathway changed significantly in different drug resistance stages. This pathway was significantly upregulated in on-treatment samples relative to baseline biopsies (Figure 3C); as tumors progressed, pathway activity was significantly suppressed in drug-resistant biopsies compared to baseline or on-treatment biopsies (Figures 3D,E). We also found that the antigen presentation pathway in drug-resistant biopsies was more active in samples with increased T cell infiltration than decreased T cell infiltration (Figure 3F). The statistical results also showed significant differences in the level of changes in the antigen presentation pathway between on-treatment and drug-resistant samples (Figure 3G). Simultaneously, correlation analysis showed that T cell infiltration abundance was highly positively correlated with the changes in the antigen presentation pathway in the process of drug resistance (Figure 3H). These data suggest that the dynamic changes in T cell infiltration abundance during MAPK-targeted therapy may be associated with altered antigen-presenting pathway activity.

### Intra-tumoral T cell infiltration is primarily regulated by major histocompatibility complex class I antigen presentation during mitogen-activated protein kinase-targeted therapy.

**FIGURE 4:**
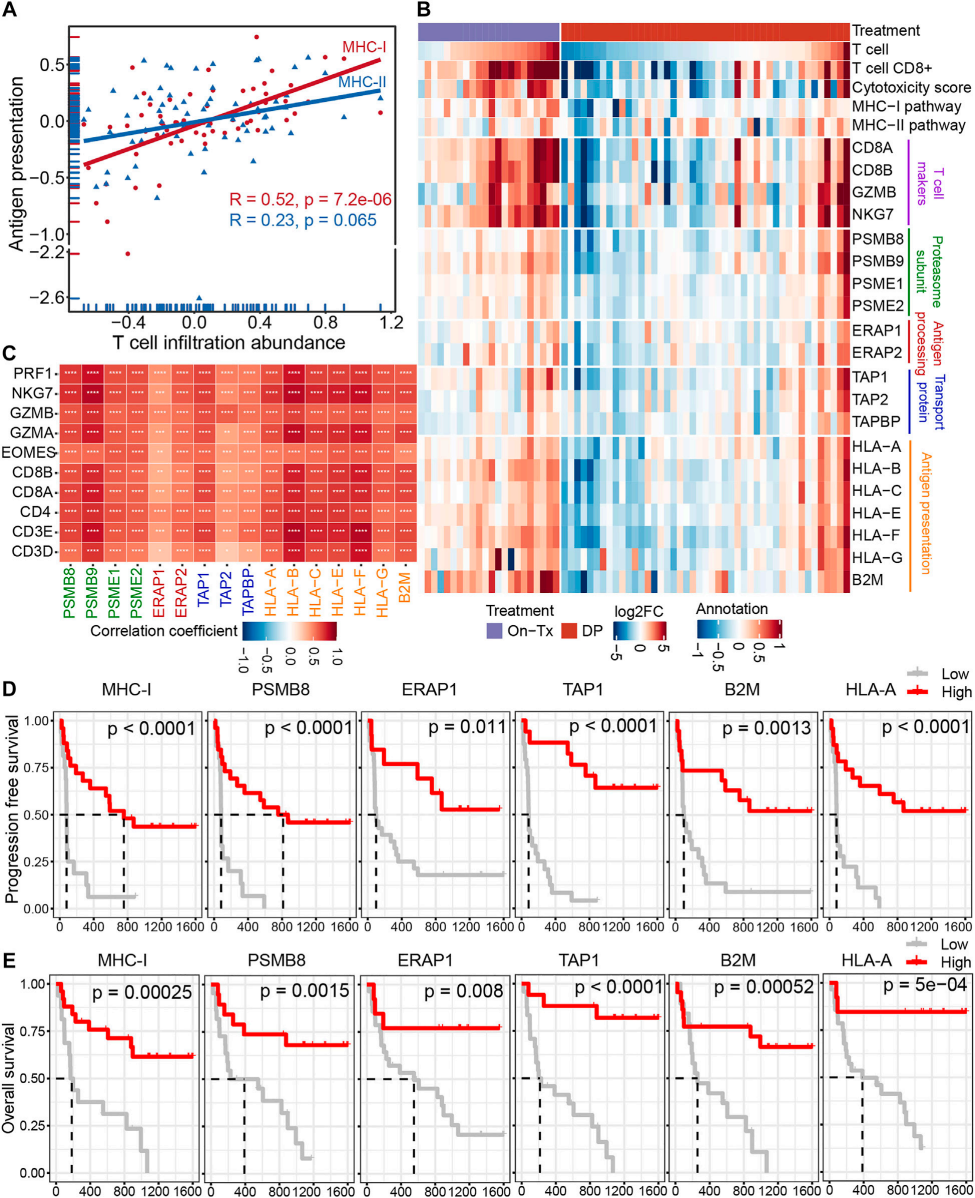
Decreased infiltration of T cells was regulated by MHC-I antigen presentation during MAPK-targeted therapy. **(A)** Pearson’s correlations between MHC class I (MHC-I) and MHC class II (MHC-II) antigen processing and presentation pathways and abundance of T cell infiltration were calculated, respectively (GSE75313, GSE65185, and EGAS00001000992). The value used to calculate the correlation was log2FC expression between MAPKi-treated and baseline biopsies. **(B)** Heatmap showing log2FC expression changes of T cell marker genes and genes involved in the four steps of MHC-I antigen processing and presentation pathway during treatment relative to before MAPKi treatment. **(C)** The tileplot shows the Pearson’s correlation of log2FC values between MHC-I molecules and T cell marker genes during MAPKi treatment. The log2FC was obtained by comparison with patient-matched baseline biopsy samples. **(D)** Kaplan-Meier plots showing the PFS of anti-PD1 treated melanoma patients, stratified by using the optimal cutoffs for the activity of MHC-I antigen presentation pathway and the expression of MHC-I molecules including PSMB8, ERAP1, TAP1, HLA-A, and B2M. Statistical tests were performed using log-rank test (PRJEB23709). **(E)** Kaplan-Meier plots showing the OS of anti-PD1 treated melanoma patients, stratified by using the optimal cutoffs for the activity of MHC-I antigen presentation pathway and the expression of MHC-I molecules including PSMB8, ERAP1, TAP1, HLA-A, and B2M. Statistical tests were performed using log-rank test (PRJEB23709). ∗*p* < 0.05; ∗∗*p* < 0.01; ∗∗∗*p* < 0.001; ∗∗∗∗*p* < 0.0001.

Antigen presentation is mediated by major histocompatibility complex class I (MHC-I) and class II (MHC-II) molecules. The activation of T cells is closely related to the presentation of tumor antigens. Neoantigens in tumor cells are delivered to the surface of tumor cells through the MHC-I antigen presentation pathway to activate and generate tumor-specific CD8^+^ T cells. The correlation analysis indicated that the expression of MHC-I molecules during MAPK-targeted therapy was more correlated with the abundance of T-cell infiltration than MHC-II antigen presentation (Figure 4A). This suggests that the down-expression of the MHC-I-mediated endogenous antigen presentation pathway is the main reason for the decline in T cell infiltration during the drug resistance. The process of presenting tumor antigen peptides to the cell surface by MHC-I through the endogenous pathway can be mainly divided into four steps: 1) the endogenous proteins are degraded by the proteasome to form antigenic peptides; 2) the antigenic peptides are transported to the endoplasmic reticulum (ER) through the transporter associated with antigen presentation (TAP); 3) further trimming of antigenic peptides by ER aminopeptidase; 4) finally, these antigenic peptides are loaded by MHC-I molecules and then transported to the cell surface (Neefjes et al., 2011). To elucidate which stage of MHC-I gene downregulation leads to inhibition of the MHC-I antigen presentation pathway during MAPK-targeted therapy. We characterized the changes in key genes involved in the four steps of the MHC-I antigen presentation pathway during MAPK-targeted therapy in melanoma biopsy transcriptome data. Unexpectedly, the expression of all these MHC-I-related genes was upregulated in on-treatment biopsies but downregulated in drug-resistant biopsies, implying that all these genes were regulated by MAPK-targeted therapy (Figure 4B). In addition, the dynamic expression of MHC-I genes coincided with the change in T-cell markers/effectors, indicating that the drug-induced immunosuppression was closely correlated with the impairment of MHC-I antigen presentation. This was confirmed through correlation analysis of these gene sets on the transcriptional level (Figure 4C). The reduction in T-cell signatures was positively correlated with the loss of MHC-I molecules, which may preclude the potential for its use in combination with T-cell based immunotherapy.

Loss of MHC-I antigen presentation is considered one of the most common mechanisms by which tumors evade host immune surveillance (Cornel et al., 2020). To further clarify the effect of MHC-I deficiency during drug resistance on the prognosis of melanoma immunotherapy. We performed a survival analysis of patients treated with anti-PD-1 immunotherapy (Figures 4D,E). The results showed that the activation of the MHC-I antigen presentation pathway and the increased expression of MHC-I related genes were associated with longer PFS and OS. These lines of evidence suggest that the poor response of MAPK-resistant melanoma to MAPK-targeted therapy combined with anti-PD-1/PD-L1 immunotherapy may be mediated by the down-expression of MHC-I antigen presentation.

**FIGURE 5:**
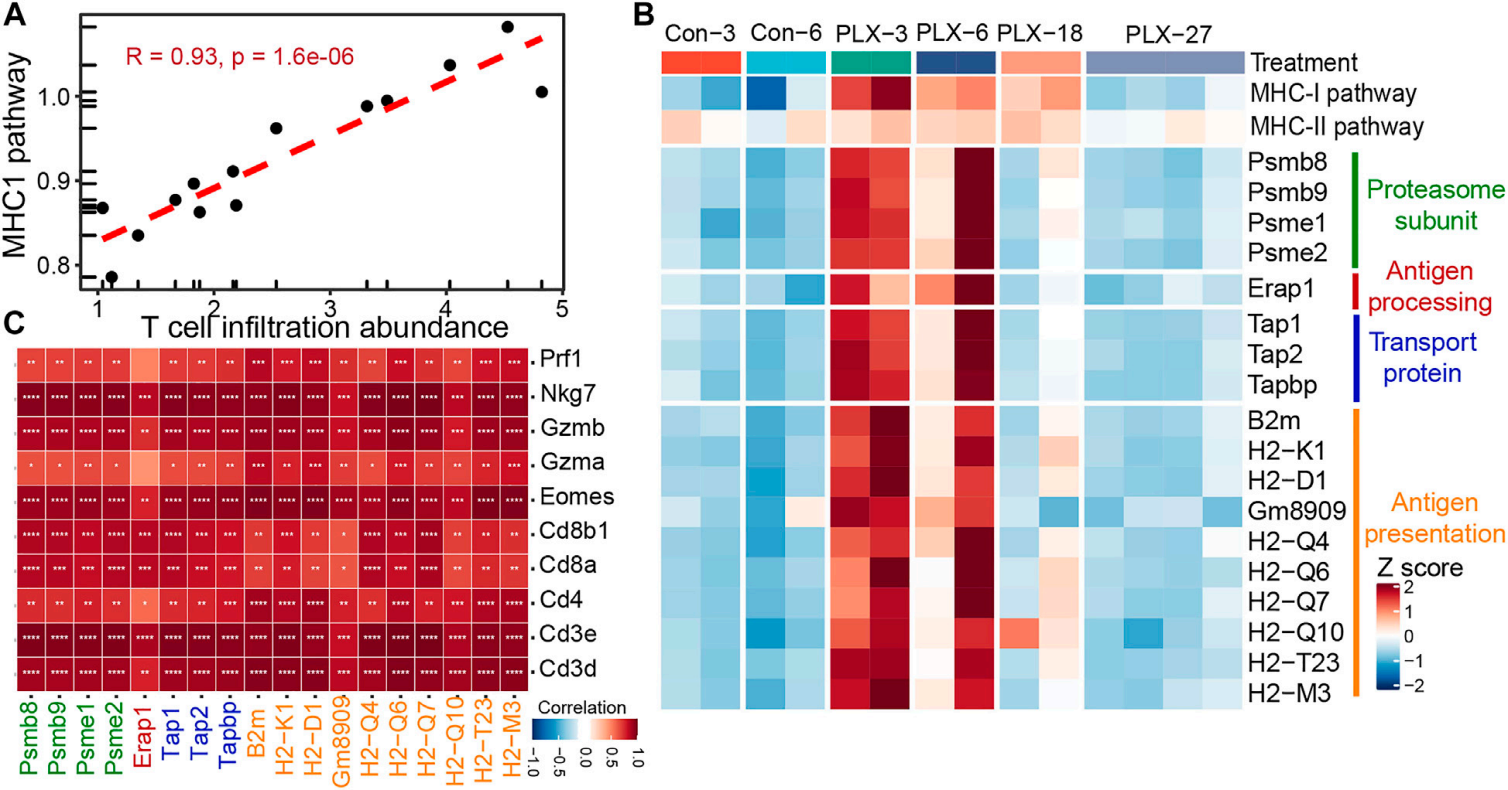
Correlation between MHC-I molecules and T cell infiltration in the mouse melanoma models. **(A)** Scatterplot showing Pearson’s correlation between MHC-I antigen processing and presentation pathways and abundance of T cell infiltration in the mouse melanoma models (GSE161430). **(B)** Heatmap showing the expression changes of MHC-I molecules at different times of BRAFi treatment in the mouse melanoma models. **(C)** Pearson’s correlations between MHC-I molecules and T cell marker genes were calculated based on the transcriptome data of the mouse melanoma models. ∗*p* < 0.05; ∗∗*p* < 0.01; ∗∗∗*p* < 0.001; ∗∗∗*p* < 0.0001.

Meanwhile, we further verified the relationship between the MHC-I antigen presentation pathway and T cell infiltration in mouse melanoma models. Consistent with our previous observations in clinical samples. The infiltrating abundance of intra-tumoral T cells exhibited a high positive correlation with the activity of the MHCI-I antigen presentation pathway during BRAFi treatment (Pearson’s correlation *r* = 0.93) (Figure 5A). All critical genes involved in the four steps of the MHC-I antigen presentation pathway were significantly upregulated in the responsive period and early drug resistance period (PLX-3/6/18) relative to the untreated group (Con-3/6). However, when progressed to the stable drug resistance period (PLX-27), the expression of these genes was significantly suppressed (Figure 5B). Furthermore, the expression levels of these MHC-I molecules and T-cell marker/effector genes showed high concordance (Figure 5C). In conclusion, intra-tumoral T cell infiltration during MAPK-targeted therapy is primarily induced by the MHC-I antigen presentation pathway, and the expression of MHC-I molecules at various stages of antigen presentation plays a crucial role in T cell infiltration.

### Down-expression of major histocompatibility complex class I genes predominantly occurred in tumor cells in resistance period.

**FIGURE 6:**
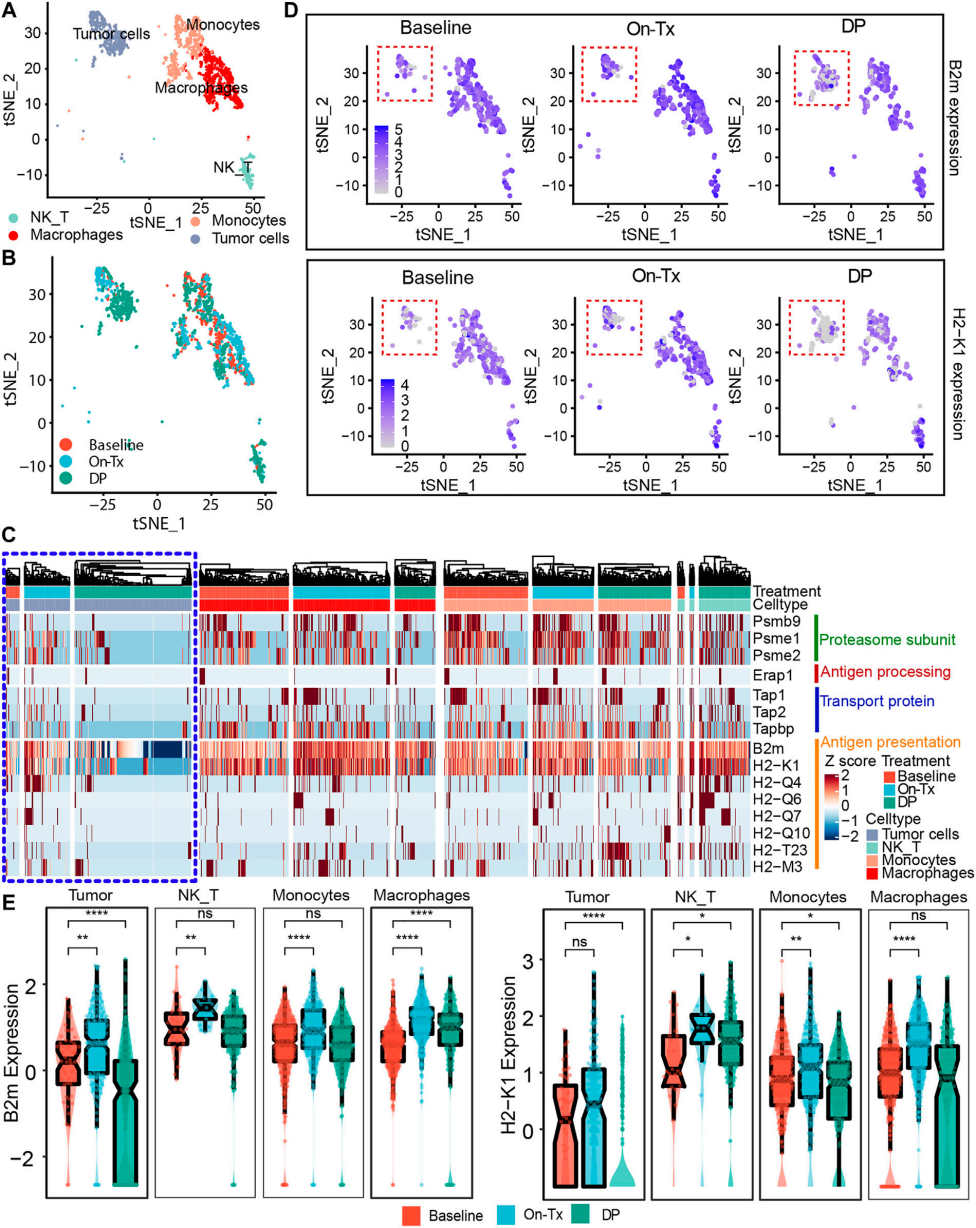
Expression of MHC-I molecules in different cell types. **(A)** t-SNE plot showing mouse single cells at different stages of the BRAFi response. Different colors indicate different cell types (GSE126714). **(B)** t-SNE plot showing mouse single cells at different stages of the BRAFi response. Different colors indicate different stages of BRAFi processing. **(C)** Heatmap showing the expression changes of MHC-I molecules in tumor cells (purple box marker), T/NK cells, macrophages, and monocytes at different stages of the BRAFi response. **(D)** Single-cell RNA-seq featurplot showing the expression of B2M and H2-K1 in baseline, On-Tx, and DP cells. Tumor cells are marked by red dotted boxes. **(E)** Single-cell RNA-seq boxplot showing the expression of B2M and H2-K1 in tumor cells, T/NK cells, macrophages, and monocytes at different stages of BRAFi response. Statistical analysis was performed using the Wilcoxon test. Baseline: cells before BRAFi; On-Tx: cells on-treatment of BRAFi; DP: cells resistant to BRAFi. ∗*p* < 0.05; ∗∗*p* < 0.01; ∗∗∗*p* < 0.001; ∗∗∗∗*p* < 0.0001.

MHC-I molecules are expressed on all nucleated cells, including tumor cells, T cells, monocytes, etc. Hence, we wanted to identify the cell types in which MHC-I expression levels were significantly altered during MAPK-targeted therapy. We analyzed the single-cell transcriptome dataset from BRAFi-treated mouse melanoma models, which contain the control (baseline), V_12d_ (vemurafenib, On-Tx), and V_Pr_ (vemurafenib, DP) samples (Long et al., 2019). This single-cell transcriptome data set defined tumor cells, T/NK cells, macrophages, and monocytes according to their canonical marker genes (Figures 6A,B). Taking advantage of these data, we checked the alteration of MHC-I levels in different cell types during MAPK-targeted therapy. We observed the expression of MHC-I molecules in tumor cells was upregulated in the drug response period and downregulated in the drug resistance period (Figure 6C). Notably, the alterations in MHC-I expression levels were mainly present in tumor cells but not in T/NK cells, macrophages, and monocytes. This phenomenon was further confirmed by the featureplot and statistical analysis, indicating that loss of MHC-I in the drug-resistant phase occurs mainly in tumor cells (Figures 6D,E). Taken together, these results support that downregulation of T cell infiltration in MAPKi-resistant tissues is primarily mediated by MHC-I loss on tumor cells.

### Identification of potential resistance-related pathways affecting the expression of major histocompatibility complex class I molecules during targeted therapy.

**FIGURE 7:**
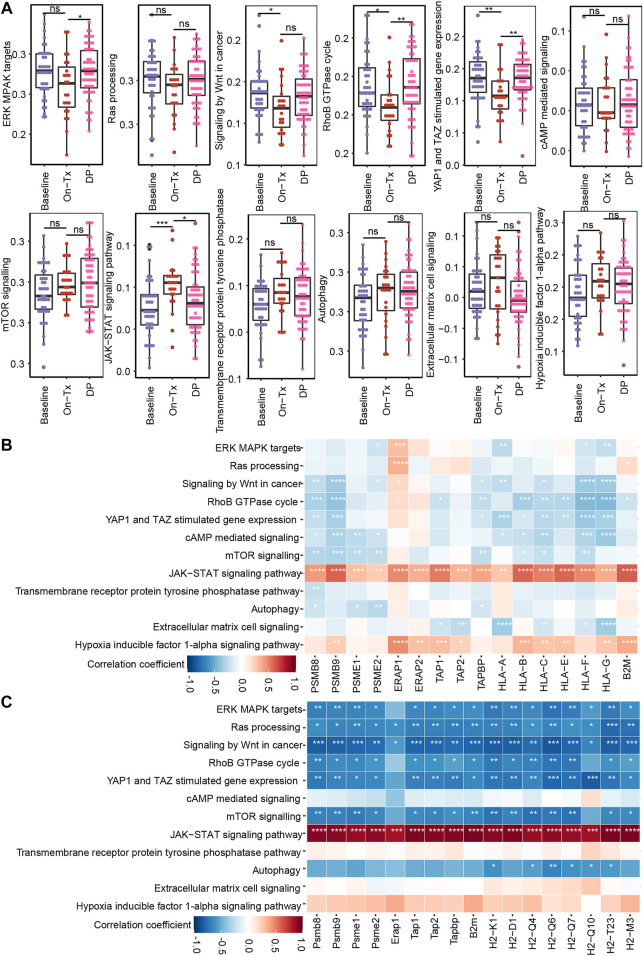
Correlation between MHC-I molecules and MAPK-targeted therapy resistance-related pathways. **(A)** Boxplot shows the activation levels of MAPKi resistance-related signaling pathways in clinical samples at different stages of targeted therapy (GSE75313, GSE65185, and EGAS00001000992). Statistical analysis was performed using the Wilcoxon test. ^ns^ *p* > 0.05; ∗*p* < 0.05; ∗∗*p* < 0.01; ∗∗∗*p* < 0.001; ∗∗∗∗*p* < 0.0001. **(B)** Heatmap showing Pearson’s correlations between MHC-I molecules and MAPKi resistance-related pathways in melanoma biopsy data (GSE75313, GSE65185, and EGAS00001000992). **(C)** Heatmap showing Pearson’s correlations between MHC-I molecules and MAPKi resistance-related pathways in the melanoma mouse models (GSE161430). ∗*p* < 0.05; ∗∗*p* < 0.01; ∗∗∗*p* < 0.001; ∗∗∗∗*p* < 0.0001.

Based on the above results, the drug resistance of tumor cells results in MHC-I molecules deficiency. We then sought to identify the potential mechanism of MAPK drug resistance related to MHC-I loss. According to previous reports, MAPK inhibition resistance in melanoma mainly occurs through the following mechanisms: 1) the reactivation of the MAPK signaling pathway; 2) compensatory activation of alternative pathways independent of MAPK, such as PI3K-mTOR, Wnt, and receptor tyrosine kinases (RTKs); 3) the activation of tumor microenvironment-related pathways, such as extracellular matrix (ECM) signaling, YAP/TAZ activity, hypoxia inducible factor 1α (HIF-1α) pathway, and ER stress-induced autophagy; and 4) some other signaling pathways, such as RhoB GTPase, cAMP, and JAK-STAT, etc., (Kozar et al., 2019; Luebker and Koepsell, 2019; Rossi et al., 2019; Yu et al., 2020; Tangella et al., 2021). By analyzing clinical samples of melanoma, we found that drug resistance-related signaling pathways exhibited staged changes during MAPK-targeted therapy. These pathways can be classified into two categories: 1) pathway activity was inhibited during the drug response period and re-activated during the disease progresses period, for example, the MAPK, Wnt, Yap, RhoB GTPase, and cAMP pathways; 2) pathway activity was compensatorily activated during the drug response period and reduced at the disease progresses period, including the mTOR, JAK-STAT, RTKs, autophagy, ECM, and HIF-1α pathways (Figure 7A). However, due to the limited sample size, the activity changes of these signaling pathways at different stages of MAPK-targeted therapy did not obtain significant statistics (*p* < 0.05) in some comparison groups.

Based on the fact that the drug resistance accompanies the loss of MHC-I antigen presentation. We speculate that the alteration of MHC-I expression levels during MAPK-targeted therapy may be related to the activation status of these drug resistance-related pathways. Consequently, we analyzed the correlation of these pathways with MHC-I molecules using clinical samples and mouse melanoma model data (Figures 7B,C). We found that MAPK, mTOR, Wnt, and RhoB GTPase exhibited significant negative correlations with MHC-I molecules. In contrast, JAK-STAT showed a positive correlation with MHC-I molecules. Interestingly, the activation of the RAS/MAPK, PI3K/mTOR pathways, and Wnt signaling pathway was reported to downregulate the expression of MHC-I molecules by inhibiting IFN or NF-κB signaling (Jongsma et al., 2021). JAK/STAT signaling is critical for MHC-I gene expression and ultimately regulates the expression of MHC-I molecules on the cell surface (Brutkiewicz, 2016). These data suggest that the dynamic expression of MHC-I-related genes during MAPK-targeted therapy may be affected by the activation status of drug resistance-related signaling pathways, such as the MAPK, mTOR, Wnt, and JAK-STAT pathways. Tumor cells inhibit MHC-I molecules expression by activating drug resistance pathways during MAPK-targeted therapy.

## Discussion

Thecombination of MAPK pathway targeted therapy and ICB is the most promising strategy for patients with advanced melanoma. However, the results of previous clinical trials showed contradictory phenomena. In the IMspire150 clinical trial, MAPK-targeted therapy and ICB therapy showed synergistic phenomena, significantly prolonging the investigator-assessed PFS of subjects (Gutzmer et al., 2020). The results of KEYNOTE-022 and COMBI-I showed that the combination of checkpoint inhibitors and targeted therapy was not significantly more effective than targeted therapy alone (Ferrucci et al., 2020; Dummer et al., 2022). Studies have also shown that anti-PD-1/PD-L1 immunotherapy is almost ineffective in patients who have previously failed BRAFi/MEKi-targeted therapy (Amini-Adle et al., 2018). To clarify the potential mechanism, we performed a systematic analysis of transcriptomic data from public datasets of baseline, on-treated, and disease progressed tumor samples from patients and mice with BRAFi or BRAFi + MEKi treatment. We found that the levels of tumor-infiltrated T cells and expression of immune-related genes were upregulated in the drug responsive period, but downregulated in the resistance period. This may explain why the combination of short-term MAPKi treatment and ICB therapy showed a synergistic effect, while ICB therapy was often ineffective in MAPK-targeted therapy-resistant samples. Through further analysis, we found that the MHC-I antigen presentation pathway was highly consistent with the changing trend of T cell abundance and function in the process of drug resistance.

MHC-I molecules play a crucial role in the adaptive immune system. Endogenous proteins are continuously degraded into oligomeric peptides *via* the ubiquitin-proteasome pathway and then bind with MHC-I molecules to form an immunogenic peptide-MHC class I (pMHC-I) complex. pMHC-I is delivered to the plasma membrane and is expressed on the surface of almost all nucleated cells (Shklovskaya and Rizos, 2021). T-cell receptors (TCRs) recognize tumor cells and mediate the activation of cytotoxic CD8^+^ T cells by interacting with pMHC-I expressed on the surface of tumor cells. Activated CD8^+^ T cells can induce tumor cell death by injecting granzymes and other cytotoxic molecules through perforin-permeabilized membranes at immunologic synapses (Taylor and Balko, 2022). Immune checkpoint inhibitors aim to relieve tumor cells against effector T cells and restore the antitumor immunity of loss-of-function T cells by blocking inhibitory immune receptors. Anti-PD-1 immunotherapy relies on the achievement of reactivation of tumor-specific CD8^+^ T cells through the presentation of melanoma neoantigens on MHC-I (Shklovskaya et al., 2020). Therefore, the success of immunotherapy depends on the recognition of tumor antigen by T cells, which in turn depends on the expression of MHC-I molecules. Previous studies have shown that the downregulation or loss of MHC-I is the mechanism of acquired resistance to anti-PD-1 therapy, which can be used as a hallmark of resistance to PD-1 inhibitors (Sade-Feldman et al., 2017; Lee et al., 2020). The downregulation of key MHC-I antigen presentation molecules is closely related to weakened antigen presentation, decreased T cells and other antitumor immune responses, and the prognosis of immunotherapy. This suggests that melanoma MAPKi-resistant cells may mediate the weakening of antigen presentation and the reduction of T cells by inhibiting the expression of MHC-I molecules, resulting in resistance to anti-PD-1/PD-L1 immunotherapy.

Downregulation or loss of MHC-I molecules does not affect cell survival, so a major mechanism by which cancer evades immune control is the loss of MHC-I antigen presentation (Dhatchinamoorthy et al., 2021). Tumors can reduce antigen presentation through several mechanisms, including genetic mutations, MHC-I diversity, epigenetic mechanisms, and transcriptional and post-transcriptional regulation (Shklovskaya and Rizos, 2021). Many oncogenic pathways have been reported to reduce antigen presentation by modulating the expression of MHC-I and related antigen-presenting components, including MAPK, PI3K/AKT, and Wnt signaling (Jongsma et al., 2021; Taylor and Balko, 2022). Through analysis, we found that the changing trend of MHC-I molecules in the process of MAPK-targeted therapy showed the opposite trend to the changes of MAPK, PI3K-mTOR, and Wnt signaling pathways. The MAPK pathway was shown to negatively regulate MHC-I by reducing the expression of IRF and STAT1 (Brea et al., 2016; Loi et al., 2016; Franklin et al., 2020). MAPK inhibitors can upregulate the mRNA expression levels of HLA-A and key molecules of the MHC-I antigen presentation pathways, including TAP1, TAP2, and β2M (Brea et al., 2016; Dhatchinamoorthy et al., 2021). Furthermore, when upstream activators of the MAPK signaling pathway are blocked, such as ALK and RET kinases, the expression of MHC-I molecules will increase (Dhatchinamoorthy et al., 2021). Activation of the PI3K signaling pathway can inhibit the induction of MHC-I molecules by IFN-γ (Chandrasekaran et al., 2019). In human head and neck squamous cell carcinoma, the IHC staining of MHC-I protein with high levels of phospho-AKT intra-tumoral regions was significantly reduced. That is, the expression pattern of phospho-AKT is opposite to that of MHC-I proteins (Chandrasekaran et al., 2019). Activation of the Wnt signaling pathway is associated with decreased MHC-I expression and c-myc-mediated HLA downregulation (Versteeg et al., 1988; Yang et al., 2020; Dholakia et al., 2022). This is consistent with the results we observed in clinical biopsies and mouse model samples, that is, during the response period of MAPK-targeted therapy, MHC-I antigen presentation increased significantly relative to baseline samples. In the drug resistance stage, the expression of MHC-I molecules was significantly inhibited due to the reactivation of the MAPK signaling pathway and the compensatory activation of alternative pathways such as PI3K-mTOR. The upregulated MAPK and PI3K-mTOR signaling pathways lead to a decrease in the expression of MHC-I molecules, which further inhibits T cell infiltration, reduces the cytotoxicity of tumor cells, and leads to immune escape. A recent study showed that durable clinical responses to anti-PD-1 immunotherapy were inversely correlated with the activity of the MAPK pathway (Fairfax et al., 2020). The finding may also explain why MAPK-resistant patients are almost ineffective in subsequent immunotherapy. Although, how these oncogenic pathways mediate the downregulation of MHC-I during MPAK-targeted therapy resistance requires further study. However, this evidence suggests that for MAPKi-resistant melanoma patients, it may be possible to promote anti-tumor immune responses by targeting the critical oncogenic signaling pathways that regulate the expression of MHC-I. To achieve synergistic anti-tumor effects of targeted therapy and immunotherapy, prolong patient survival, and improve patient prognosis.

Our work systematically analyzes the alteration of the tumor immune microenvironment in clinical biopsies and mouse models during targeted therapy. We found that T cells infiltration and immune-related gene expression were upregulated in on-treatment tumors and downregulated in disease-progression tumors. This suggests that the tumor immune microenvironment was agitated by MAPK targeted therapy and correlated with drug response status. Moreover, the alteration of tumor-infiltrating T cells was mainly regulated by the expression level of MHC-I molecules on the surface of tumor cells. During MAPK-targeted therapy, tumor cells activate drug resistance-related pathways, such as the MAPK, PI3K-mTOR, and Wnt pathways, which results in the loss of MHC-I molecules and dampening the antitumor immune response. This phenomenon can explain why targeted therapy-resistant patients had lower response rates to immunotherapy. Taken together, our results demonstrated that the introduction of immune checkpoint inhibitors during the response period of targeted therapy may be superior to drug resistance period. And for patients who have acquired resistance to targeted therapy, the combination of ICB with inhibitors of MAPKi-resistant pathways is a potential treatment option.

## Data Availability

The datasets presented in this study can be found in online repositories. The names of the repository/repositories and accession number(s) can be found in the article.
